# Papillary-Cystic Variant of Acinic Cell Carcinoma in the Lacrimal Gland

**DOI:** 10.1155/2014/285485

**Published:** 2014-05-15

**Authors:** Miles Bannister, Greig Lawson

**Affiliations:** Department of Otolaryngology, Head & Neck Surgery, Aberdeen Royal Infirmary, Foresterhill Road, Aberdeen AB25 2ZN, UK

## Abstract

Papillary-cystic variant of acinic cell carcinoma is a rare tumour confined to salivary gland tissue. Here, we report the first case ever to manifest in a tumour affecting the lacrimal gland, a completely different tissue type, and review the current published literature on this type of tumour.

## 1. Introduction

Papillary-cystic variant is a very rare form of acinic cell carcinoma. The handful of cases reported in the literature have manifested mainly in salivary gland tissue, affecting both the major and minor salivary glands. The tumour type behaves in a similar manner to other varieties of acinic cell carcinoma by presenting as a slowly enlarging mass within normal tissue. In this paper, we describe a manifestation of papillary-cystic variant of acinic cell carcinoma (PCV-ACC) presenting as a mass within the right lacrimal gland and discuss the management and arrangements for patient review. This is one of only a few tumours presenting in the lacrimal gland.

## 2. Case Report

A 78-year-old lady presented to our service in February 2013 with a 12-month history of a mass over the right eye. The mass had been gradually enlarging but was not associated with any pain or change in periocular sensation. She denied any vision loss, double vision, epiphora, or xerophthalmia. The patient suffered from hypertrophic cardiomyopathy with an implantable defibrillator inserted to maintain cardiac function but was otherwise fit and well.

**Figure 1:**
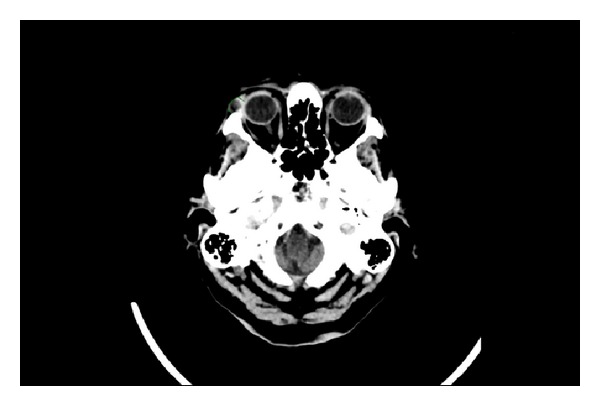
Axial view of CT scan showing a right supraorbital mass prior to surgery.

On examination, a firm mass was arising from the palpebral lobe of the right lacrimal gland, underneath the supraorbital rim. Visual field testing and ophthalmoscopy examination were normal. Visual acuity testing using a Snellen chart revealed 20/70 vision in both eyes, which was in a normal range for a person of this age. Cranial nerve, ear, nose, and throat examinations were all normal. A computed tomography (CT) scan reported that the mass was a soft tissue tumour arising from the palpebral lobe of the lacrimal gland but that the orbits and globes were otherwise normal with no tissue compression or invasion (Figure 1).

The entire right lacrimal gland was removed in our hospital under general anaesthetic through an orbitotomy. The expectation was that the tumour would be a pleomorphic adenoma and that removal would prevent future carcinoma development requiring more aggressive surgery with loss of vision. Following surgery the patient made a good recovery and was discharged from hospital the day after surgery. Hypromellose 0.3% eye drops were provided for the right eye to compensate for the reduced lacrimation that would now affect that eye.

**Figure 2:**
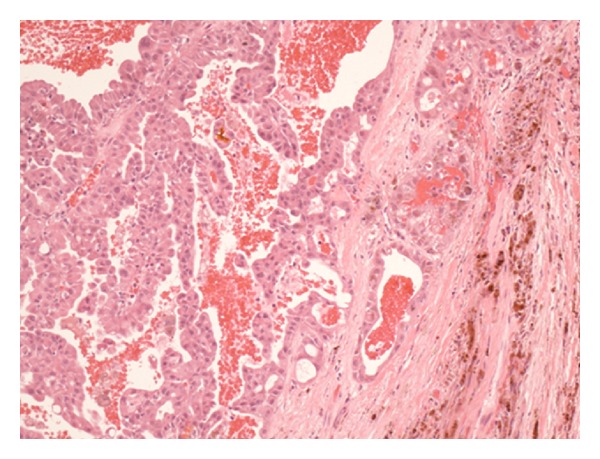
The microscopic examination shows cystic characteristics.

**Figure 3:**
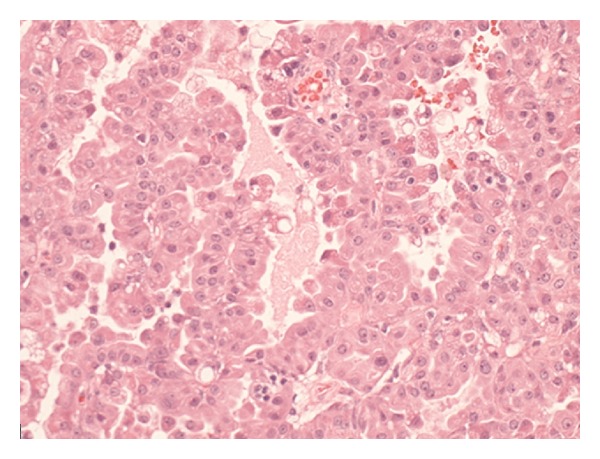
The microscopic examination shows atypical acinar architecture.

**Figure 4:**
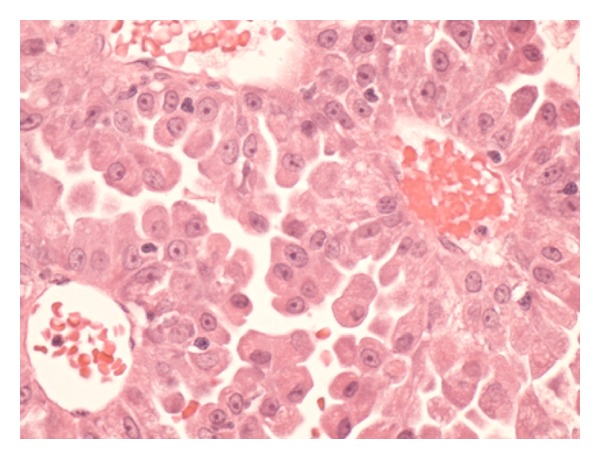
The microscopic examination shows rounded, polygonal cells.

Histopathological assessment of the removed gland revealed that the soft tissue mass within the lacrimal gland was a PCV-ACC (Figures 2, 3, and 4). Analysis of the specimen did reveal that the carcinoma had been excised in its entirety with sufficient margins of normal tissue surrounding the carcinoma.

The patient's case was discussed at our local head and neck cancer multidisciplinary team meeting. When the histological subtype was considered, the meeting advised that a chest X-ray and ultrasound scan of the liver should be performed to assess pulmonary or liver metastases, respectively. Both these investigations were normal and the patient remains under review by our service. No further complications of surgery developed and to date no recurrence or new tumour has developed.

## 3. Discussion

Despite being histologically similar, PCV-ACC principally presents in the salivary glands [1]. PCV-ACC was first identified in 1994 [2]. Currently it is classified as one of a number of low-grade salivary duct carcinomas, which include cystadenoma, cystadenocarcinoma, sclerosing polycystic adenosis, oncocytoma, and salivary duct carcinoma in situ [3]. A diagnosis is difficult based on fine needle aspiration cytology alone [4, 5]. The cystic nature of the tumour dilutes the cellularity of the sample that is obtained and the cytoarchitecture is different from acinic cell carcinoma [4]. The PCV-ACC cells themselves are also different in appearance from acinic cell carcinoma by having a polygonal appearance that can mimic clear-cell carcinoma [4]. The cystic nature can lead to misinterpretation of a mucin background and misdiagnosis as cystadenocarcinoma [5, 6]. The rare nature of the tumour can lead to misdiagnosis too, with samples misinterpreted as being commoner tumours [1, 6]. Therefore, histological diagnosis is preferred [3].

Sheyn et al. suggested that PCV-ACC presents in the elderly, though Shet et al. later reported a series of exclusively young patients with the tumour [1, 7]. No articles have been published on the long-term prognosis of patients diagnosed with PCV-ACC or on any specific treatments that are different from those used to treat acinic cell carcinoma. Even acinic cell carcinoma is uncommon in the lacrimal gland with very few reports of the tumour developing at this site [8]. The large series of lacrimal gland malignant tumour types published by Wright et al. revealed that adenoid cystic carcinoma and carcinoma ex pleomorph were the two commonest types accounting for 76% and 12% of malignancies, respectively [9]. Pleomorphic adenoma remains the commonest overall neoplasm of the lacrimal gland [10].

In conclusion, although PCV-ACC is an extremely rare carcinoma, it should be considered in the differential diagnosis of lacrimal gland tumours because the additional investigations required are so different from that provided for the common lacrimal gland carcinomas. Additionally, head and neck surgeons should be aware of the presence and behaviour of PCV-ACC in salivary glands.
